# Isolation and Identification of Phosphate-solubilizing Bacteria in the Rhizosphere of *Robinia pseudoacacia* on the Loess Plateau and Verification of Phosphate Solubilization Capacity

**DOI:** 10.1264/jsme2.ME24001

**Published:** 2024-09-14

**Authors:** Wenrui Zhang, Yuhao Zhou, Jingru Jia, Yinjun Lu, Haoqiang Zhang

**Affiliations:** 1 College of Forestry, Northwest A&F University, Yangling, Shaanxi 712100, China

**Keywords:** phosphate-solubilizing bacteria, *Robinia pseudoacacia*, the Loess Plateau

## Abstract

The Loess Plateau is one of the key areas for soil and water erosion control in China. Planting vegetation, such as *Robinia pseudoacacia*, is one of the mainstream methods to prevent soil and water erosion. However, the combination of abundant calcium ions and phosphate in the soil of the Loess Plateau limits the phosphorus nutrition of plants. In the present study, soil samples were collected under the *R. pseudoacacia* forest, from which two PSB strains with efficient phosphate solubilization capacities, named PSB2 and PSB7, were isolated and screened. The dissolved phosphate concentrations of their culture media were 9.68-fold and 11.61-fold higher, respectively, than that of the control group. After identification, PSB2 was classified as *Pseudomonas* and PSB7 as *Inquilinus*. This is the first time that *Inquilinus* has been isolated as a PSB from calcareous soil in the Loess Plateau. We then investigated the effects of different growth conditions on their phosphate solubilization capacities. Both strains effectively utilized glucose and ammonium nitrogen while maintaining high phosphate solubilization efficiency. In addition, PSB2 preferred to survive under neutral conditions and PSB7 under acidic conditions. Pot experiments indicated that the inoculation with PSB7 significantly increased the phosphorus content in the roots of *R. pseudoacacia*. These results imply the potential of this PSB as a phosphorus biofertilizer for *R. pseudoacacia*, which may be beneficial for soil and water management on the Loess Plateau.

The Loess Plateau is one of the key areas for soil and water erosion control in China, and the method of planting vegetation has been widely utilized in numerous control strategies (Yu *et al.*, 2016). However, the low level of soluble phosphate in the soil inhibits plant growth (Zhu *et al.*, 2012), which is due to the abundance of calcium ions in the Loess Plateau soil that bind with soluble phosphate, resulting in a low level of phosphorus available in the soil for absorption by plants (Zhang *et al.*, 2005; Harris *et al.*, 2006). Phosphorus is one of the macronutrients required by plants and participates in metabolic processes in organisms of various forms (Raghothama, 1999; Vance *et al.*, 2003). A number of strategies have been employed to overcome phosphorus deficiency in soil. A previous approach that was widely implemented in fields involved using large amounts of chemical fertilizers; however, this resulted in the eutrophication of groundwater and runoff water, creating ecological pollution (Bayer *et al.*, 2001; Sharma *et al.*, 2013). Against this background, a ecofriendly strategy involving the addition of phosphate-solubilizing bacteria (PSB) to soil in order to increase available phosphate levels has been applied.

PSB are a type of rhizosphere microorganism that exert positive effects on plants and mainly include *Pseudomonas*, *Acinetobacter*, *Bacillus*, *Burkholderia*, *Klebsiella*, and *Pantoea* (Panwar *et al.*, 2014; Nagargade *et al.*, 2018). The substances they secrete during growth, such as organic acids, inorganic acids, phosphatases, and phytases, dissolve phosphorus that has already precipitated and bound with other metal ions in the soil (Miller *et al.*, 2010; Roy and Roy, 2019). PSB have been applied as a biological phosphorus fertilizer to improve the nutritional aspects of economic crops. Differing from its widespread application in agriculture, basic research on and the application of PSB in the context of ecological restoration are limited (Khan *et al.*, 2007; Sharma *et al.*, 2013; Chen *et al.*, 2021). Hameeda *et al.* (2006) found that in addition to the growth environment, the species of PSB was an important factor affecting phosphate solubilization capacities. Indigenous strains are more effective at ameliorating local phosphorus deficiencies because they are better adapted to the specific environment. Therefore, the isolation and identification of PSB from the Loess Plateau soil are crucial in this study.

*Robinia pseudoacacia* possesses fast-growing and drought-resistant characteristics, making it one of the primary trees planted for soil and water conservation in the Loess Plateau (Qiu *et al.*, 2010; Xu *et al.*, 2014). The growth of trees is restricted by a lack of soluble phosphate in the soil. Although the soluble phosphate level available for plants is low, phosphorus is present in Loess Plateau soil, which is bound to calcium ions and precipitated. In theory, the addition of PSB to the soil may dissolve these precipitates, thereby releasing available phosphate and alleviating the deficiency of phosphorus in this region, which will promote plant growth and, thus, accelerate the pace of soil and water conservation.

In the present study, we collected soil samples from the rhizosphere of *R. pseudoacacia* in the Loess Plateau with the aim of isolating PSB to produce available phosphorus in the soil. The PSB species identified were then exami­ned to investigate the effects of different growth conditions on their phosphate solubilization capacities. A pot experiment was conducted to confirm whether the addition of PSB indirectly increased plant phosphorus absorption and improved phosphorus nutrition.

## Materials and Methods

### Soil sample collection

Soil samples were collected from the *R. pseudoacacia* forest (35°12′36″N, 107°41′24″E) in Hongjia town, Changwu county, Xianyang city, Shaanxi Province. Forest soil was not fertilized for 10 years. Soil had a pH of 8.72 (1:5, soil:water [w/v]) and contained 0.07‍ ‍g (kg total nitrogen)^–1^, 0.25‍ ‍g (kg total phosphorus)^–1^, and 5.40‍ ‍g (kg total potassium)^–1^. The three-point sampling method was used to collect the rhizosphere soil of *R. pseudoacacia* at a depth of 20–30‍ ‍cm. At the same time, some capillary roots and adhered soil were placed into sterile plastic bags. All samples were stored in a refrigerator at 4°C for later ana­lyses and isolation.

### Separation and purification of candidate strains

The gradient dilution method was used to isolate PSB on the selective medium NBRIP (Nautiyal, 1999). This medium contained the following (L^–1^): 10‍ ‍g of glucose, 0.5‍ ‍g of (NH_4_)_2_SO_4_, 0.3‍ ‍g of NaCl, 0.3‍ ‍g of KCl, 2.0‍ ‍g of Ca_3_(PO_4_)_2_, 0.3‍ ‍g of MgCl_2_·4H_2_O, 0.3‍ ‍g of MgSO_4_·7H_2_O, 0.03‍ ‍g of FeSO_4_·7H_2_O, 0.03‍ ‍g of MnSO_4_·4H_2_O, and 20‍ ‍g of agar powder, and pH was adjusted to 7–7.3. Tricalcium phosphate was used as the phosphorus source in the medium because phosphorus precipitation in the Loess Plateau primarily exists in the form of calcium phosphate.

The preliminary isolation of strains on NBRIP plates was performed using the concentration gradient dilution method described by Sharma *et al.* (2013). We selected single colonies with transparent zones and transferred them to NBRIP plates for purification and cultivation. The presence of transparent zones around the colonies is an important indicator of the phosphate solubilization capacity of bacteria. Once new colonies grew, we checked whether their characteristics matched the previous colonies and observed them under a microscope to confirm if they were single microorganisms. If contaminants are found, these bacteria were re-isolated and purified until a pure culture was obtained.

### Screening PSB with efficient phosphate solubilization capacities

The observation of a transparent circle around colonies is a qualitative method for assessing the phosphate solubilization capacity of PSB. To further quantify this, we employed the ammonium molybdate spectrophotometric method described by Paul and Sinha (2017). The selected strains from the previous step were cultured in LB medium to a concentration of 10^9^ CFU mL^–1^. Five hundred microliters of the culture was then transferred to 50‍ ‍mL of NBRIP liquid medium and cultured at 28°C with shaking at 180‍ ‍rpm. LB medium was used as a control group instead of the bacterial culture with three replicates for each strain. The measurement of soluble phosphate concentrations in the culture medium of each strain was conducted after 6 days. We simultaneously measured the pH of the supernatant of the culture medium.

### Identification of PSB

Bacteria were identified using Gram staining and 16S rRNA gene sequencing. After Gram staining, bacteria were observed under a microscope and their color and morphology were recorded. The DNA of the strain was extracted with the DNA Extraction Kit (Omega Bacterial DNA kit 200). 16S rRNA gene was amplified by PCR with the bacterial universal primers 27F-5′-AGAGTTTGATCCTGGCTCAG-3′ and 1492R-5′-GGTTACCTTGTTACGACTT-3′ (Weisburg *et al.*, 1991). Amplification was performed by initial denaturation at 95°C for 3‍ ‍min, followed by 35 cycles at 95°C for 15‍ ‍s, 56°C for 30‍ ‍s, and 72°C for 1‍ ‍min, with a final extension at 72°C for 5‍ ‍min. PCR products were sent to Sangon Biotech (Shanghai) for sequencing. The sequences obtained in the present study were analyzed by BLAST and compared with the corresponding sequence in the GenBank database. We used the neighbor-joining method to construct the phylogenetic tree in MEGA11 software (Tamura *et al.*, 2021). We requested accession numbers in the database GenBank for nucleic acid sequences. The accession numbers for the nucleic acid sequences of PSB2 and PSB7 in the database GenBank were OP050166 and OP050167, respectively.

### Effects of growth conditions on the amount of phosphate solubilized by PSB

Strains PSB2 and PSB7, which were screened in previous experiments, were selected as the research objects in this experimental step. To investigate the effects of growth conditions on the‍ ‍amount of phosphate solubilized by PSB, strains PSB2 and PSB7 were cultured in modified NBRIP medium. This involved changing the carbon source, nitrogen source, carbon-to-nitrogen ratio (C/N), and initial pH of the medium. By maintaining equal molar amounts of carbon and nitrogen in the culture medium, we‍ ‍calculated their masses. Glucose (10.00‍ ‍g‍ ‍L^–1^), maltose (9.09‍ ‍g‍ ‍L^–1^), lactose (8.63‍ ‍g‍ ‍L^–1^), sucrose (8.63‍ ‍g‍ ‍L^–1^), and soluble starch (8.18‍ ‍g‍ ‍L^–1^) were utilized as alternative carbon sources. As the nitrogen source, (NH_4_)_2_SO_4_ (0.50‍ ‍g‍ ‍L^–1^), NaNO_3_ (0.64‍ ‍g‍ ‍L^–1^), NH_4_CNO (0.23‍ ‍g‍ ‍L^–1^), and NH_4_NO_3_ (0.30‍ ‍g‍ ‍L^–1^) were used. Glucose and ammonium nitrate served as carbon and nitrogen sources, respectively. C/N was affected by adjusting the content of nitrogen added while maintaining the carbon content at 1% (w/v). Four initial C/N was set: 60:1, 40:1, 25:1, and 10:1. The initial pH of media were adjusted to 4, 5, 6, 7, 8, and 9 using HCl and NaOH. Three replicates for each treatment were prepared. LB culture medium was used as the control group instead of the bacterial inoculation. Bacteria were cultivated at 28°C and 180‍ ‍rpm for 6 days. Soluble phosphate concentrations and pH in the culture media of the strains were then measured.

### Pot experiment

After air-drying the soil collected from the forest, it was sieved through a 2-mm sieve and autoclaved at 121°C for 3‍ ‍h to serve as the potting substrate. To ensure the effectiveness of PSB, 1‰ (w/w) tricalcium phosphate was added and mixed into the substrate.

*R. pseudoacacia* seeds were surface disinfected with 5% sodium hypochlorite for 10‍ ‍min and washed with sterile water five times. Seeds were then immersed in sterile water at 60°C and left to cool to room temperature overnight. Surface-sterilized seeds were placed in Petri dishes with sterilized filter paper, and the paper was moistened for cultivation at 28°C. After a few days, germinated seeds were transplanted into 50-hole seedling pots containing sterilized nutrient-free sand and vermiculite ([v:v], 1:1). To maintain the normal growth of seedlings, 5‍ ‍mL of P-containing Hoagland nutrient solution was added per week to each hole. After three weeks, seedlings with consistent growth were selected and transferred to a pot containing 300‍ ‍g of soil. Three seedlings were planted in each pot, and after two weeks, seedlings were thinned to one per pot. Only water was supplied daily.

PSB2 and PSB7 cultured for 36‍ ‍h were used as the inocula. The supernatant was discarded after centrifuging the bacterial culture, and the remaining cells were resuspended in sterile water. The cell concentration was adjusted to approximately 10^9^ CFU mL^–1^ for later use. Six weeks after transplanting, 3‍ ‍mL (1%, v/w) of the PSB inoculum was added to the soil, with the addition of sterile water as the control group. The experiment consisted of three treatments: no inoculum (CK), the PSB2 inoculum (PSB2), and the PSB7 inoculum (PSB7). There were five replicates for each treatment. The experiment was conducted in a greenhouse with a 16-h/8-h light/dark cycle at a temperature of 24–28°C and relative humidity of 40–60%.

### Assessment of indicators

Eight weeks after the inoculation with PSB, samples were collected by separating the shoots and roots, followed by weighing. Parts of the shoots and roots were dried at 65°C until they reached a constant weight and were then subjected to measurements of the content of phosphorus. The phosphorus content of the plant was assessed using the molybdenum blue color method after sample grinding and digesting (Watanabe and Olsen, 1965). Potted soil was sampled to measure the concentration of soluble phosphate and alkaline phosphatase activity.

Soil phosphorus was leached and filtered using phosphorus-free activated carbon and 0.5‍ ‍mol‍ ‍L^–1^ sodium bicarbonate leachate (pH=8.5). The filtrate was then used to evaluate the effective phosphorus content of the soil using the molybdenum-antimony colorimetric method (Watanabe and Olsen, 1965). Fresh soil samples were mixed with toluene and 0.5% sodium phosphate buffer and incubated at 37°C for 24 h. At the end of the incubation, 0.3% aluminum sulphate solution was added and filtered through filter paper. The filtrate was used to measure alkaline phosphatase activity in the soil using the disodium phosphate colorimetric method (Tabatabai and Bremner, 1969). A soil-free control was set up for each experiment.

### Data ana­lysis

Data were subjected to an ana­lysis of variance (ANOVA) and differences among treatments were detected by Tukey’s test (*P*<0.05) for the amount of phosphate solubilized by bacteria and plant assays. In all cases, SPSS 20.0 software was used.

## Results

### Screening and isolation of PSB

**Fig. 1.**
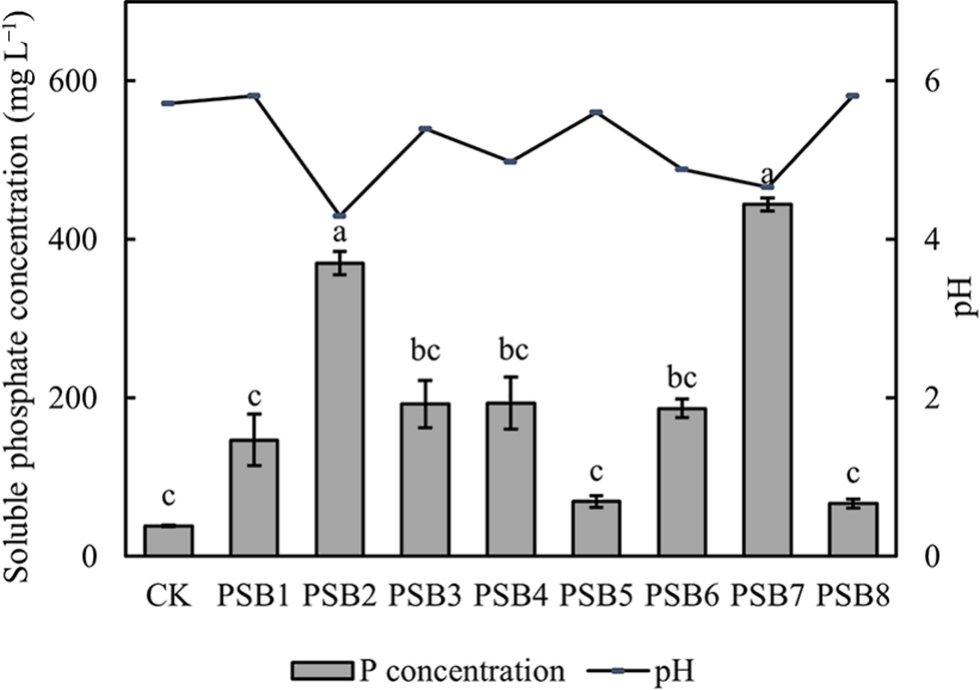
Soluble phosphate concentration after the cultivation of initially screened strains. Data are presented as means±SD. According to Tukey’s test, the same letters in the same column indicate no significant differences among treatments at a 0.05 level (*n*=3).

After a 3-day culture on NBRIP plates, eight colonies with distinct transparent zones were screened out, purified, and named PSB1 to PSB8. A significant amount of phosphate was released by the inoculations with PSB2 and PSB7. The amounts of phosphate released were 370.11‍ ‍mg‍ ‍L^–1^ by PSB2 and 443.99‍ ‍mg L^–1^ by PSB7, which were 9.68-fold and 11.61-fold higher, respectively, than that of the control group. Their supernatants also showed the lowest pH (Fig. 1).

### Identification of strains

**Fig. 2.**
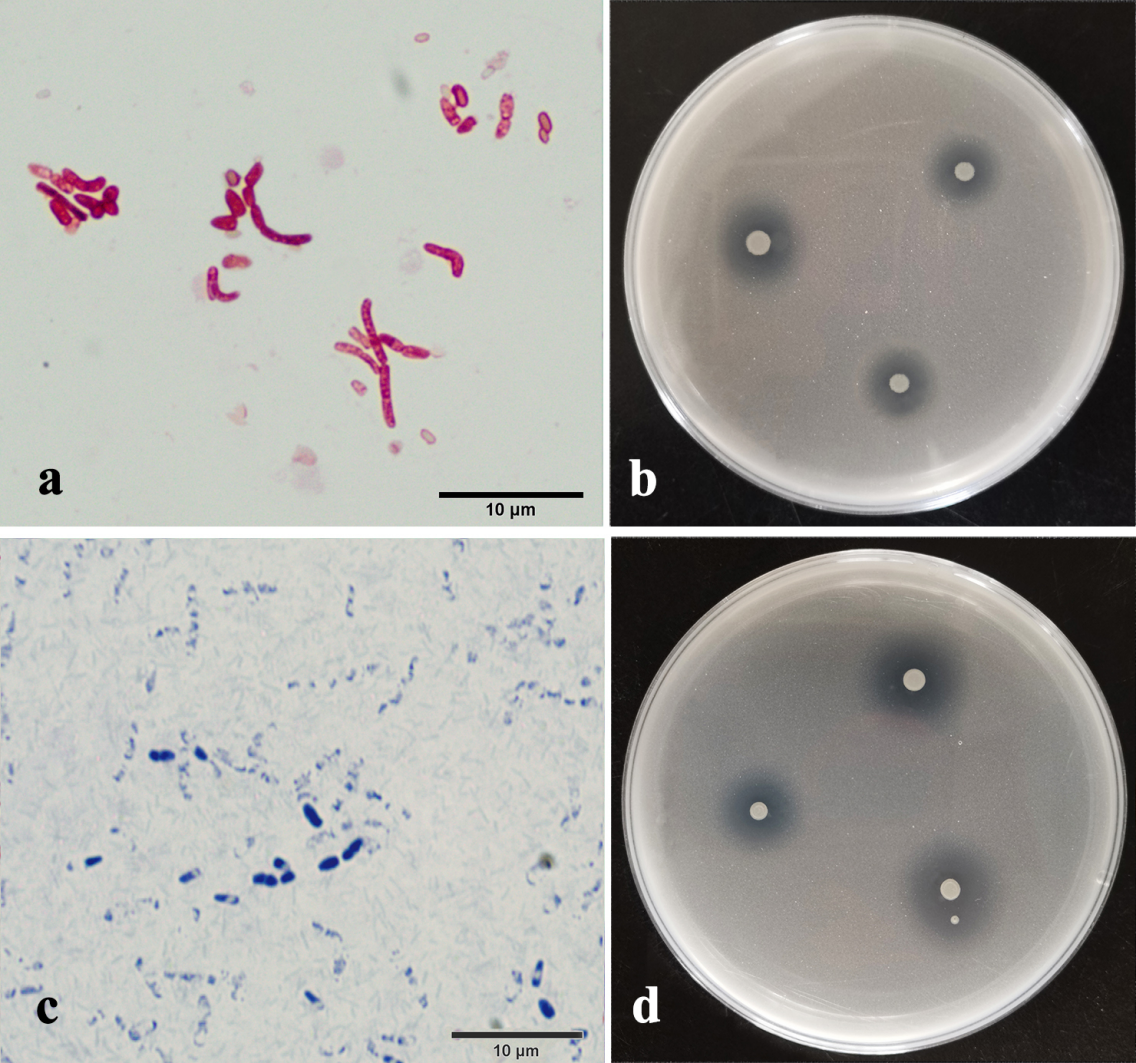
Morphological characteristics of strains. PSB2 (a) and PSB7 (c) were identified as Gram-negative and Gram-positive bacteria, respectively. The dissolved phosphorus halo of isolates on NBRIP plates, b for PSB2, d for PSB7.

**Fig. 3.**
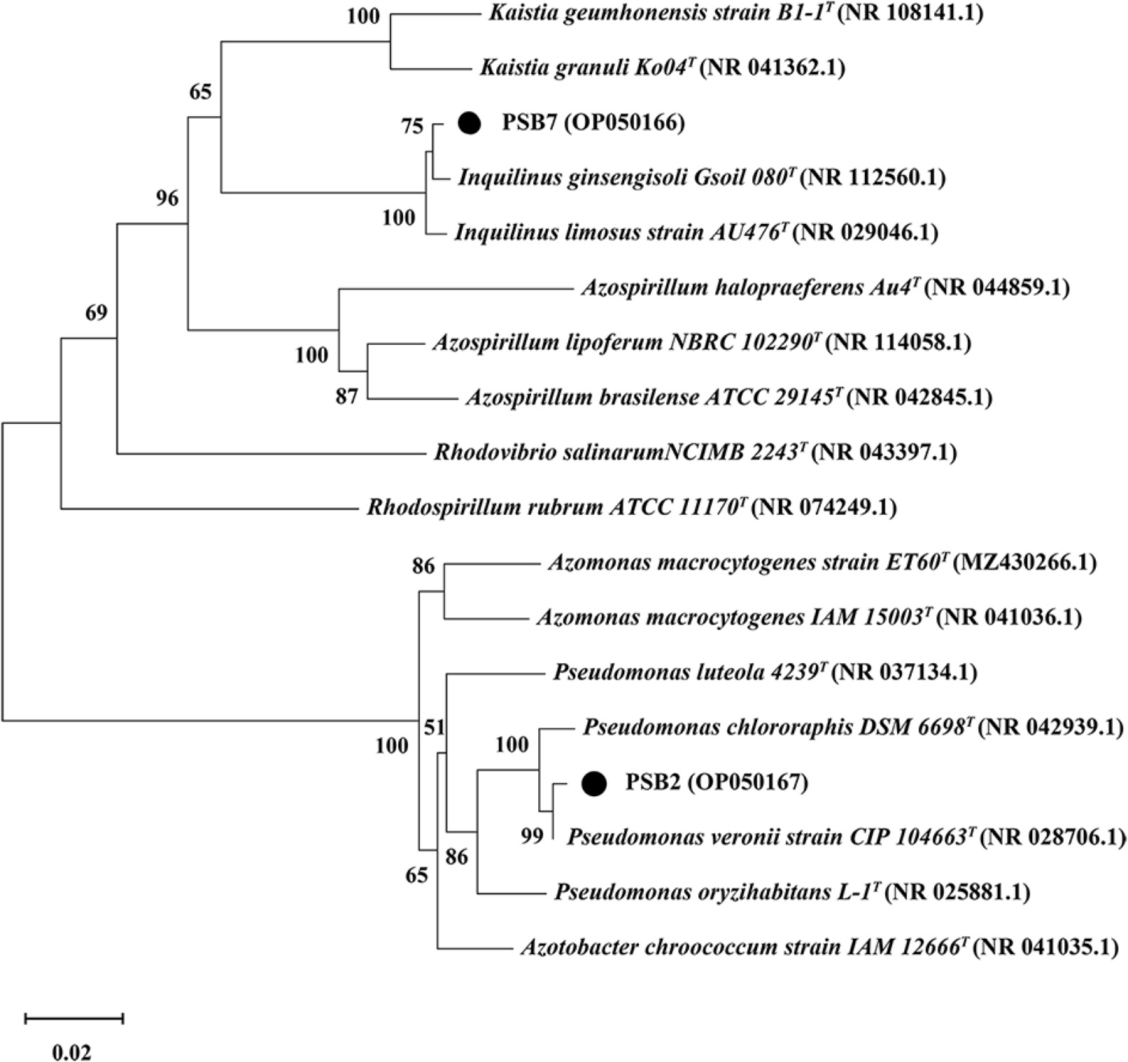
Phylogenetic tree of PSB2 and PSB7. A neighbor-joining tree based on 16S rRNA gene sequences showing the phylogenetic positions of the strains.

PSB2 was a Gram-negative bacterium with a long rod shape. After 3 days of growth, the colony appeared translucent white, with a rough surface and an overall irregular oval shape (Fig. 2a and b). The 16S rRNA gene sequence showed that PSB2 had high similarity with *Pseudomonas veronii* and *Pseudomonas chororaphis*. Fig. 3 shows the phylogenetic tree, in which PSB2 appeared with the accession number OP050167, related to *P. veronii* and *P. chororaphis*. PSB7 was a Gram-positive bacterium with a short rod shape. After 3 days of growth, the colony appeared milky white, with a smooth, slight surface and an overall irregular oval shape (Fig. 2c and d). The 16S rRNA gene sequence showed that PSB7 had high similarity with *Inquilinus ginsengisoil* and *Inquilinus limosus*. Fig. 3 shows the phylogenetic tree in which PSB7 appeared with the accession number OP050166, related to *I. ginsengisoil* and *I. limosus*.

### Phosphate solubilization characteristics

**Fig. 4.**
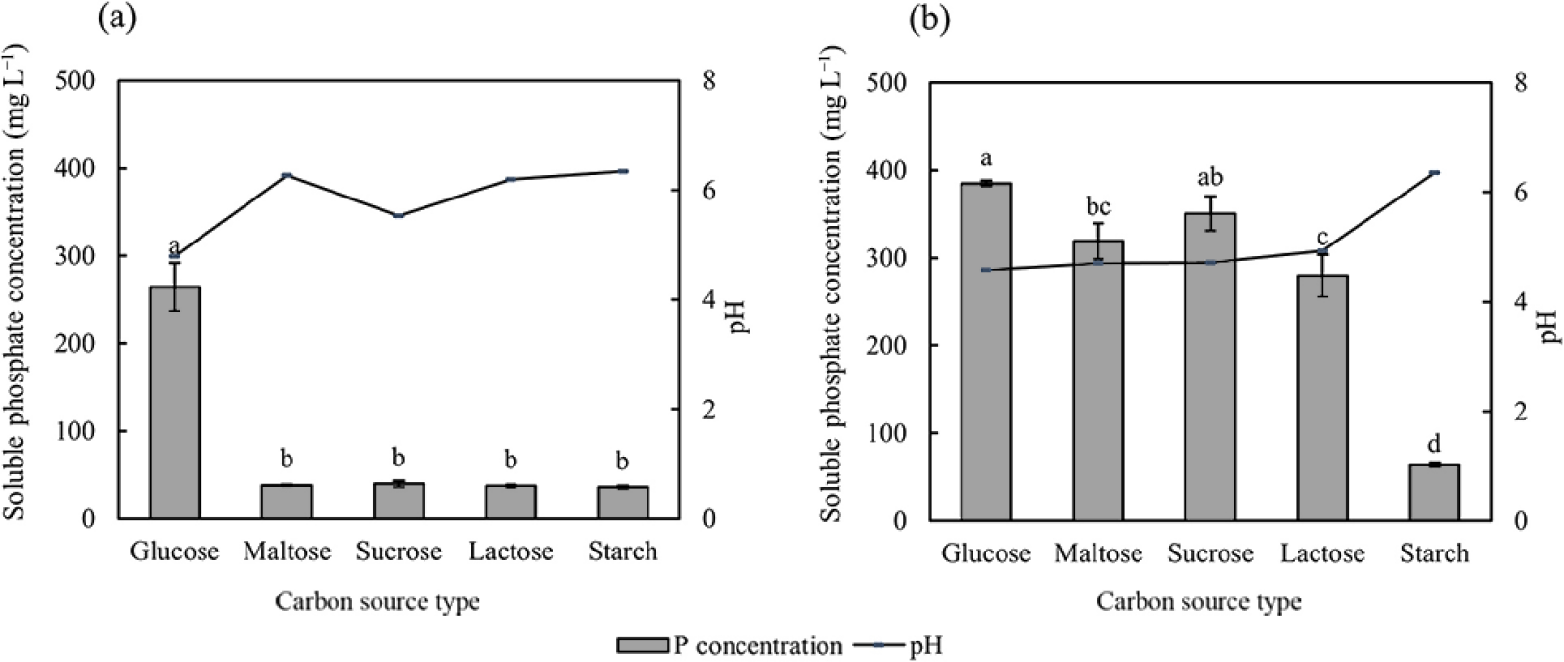
Effects of the carbon source on soluble phosphate concentrations after the cultivation of PSB2 (a) and PSB7 (b). The value of soluble phosphate shown in the figure is the amount of soluble phosphate in the control group that has been subtracted. Data are presented as means±SD. According to Tukey’s test, the same letters in the same column indicate no significant differences among treatments at a 0.05 level (*n*=3).

The carbon source affected the amount of phosphate solubilized by PSB (Fig. 4). When glucose was used as the carbon source, PSB2 and PSB7 both exhibited high phosphate solubilization capacities, with soluble phosphate concentrations in media of 264.43 and 385.05‍ ‍mg L^–1^, respectively. The pH of the medium was significantly lower than that in the other treatment groups. According to the results obtained, PSB7 maintained a better phosphate solubilization capacity when the carbon source varied, except for soluble starch. However, PSB2 did not effectively utilize carbon sources other than glucose.

**Fig. 5.**
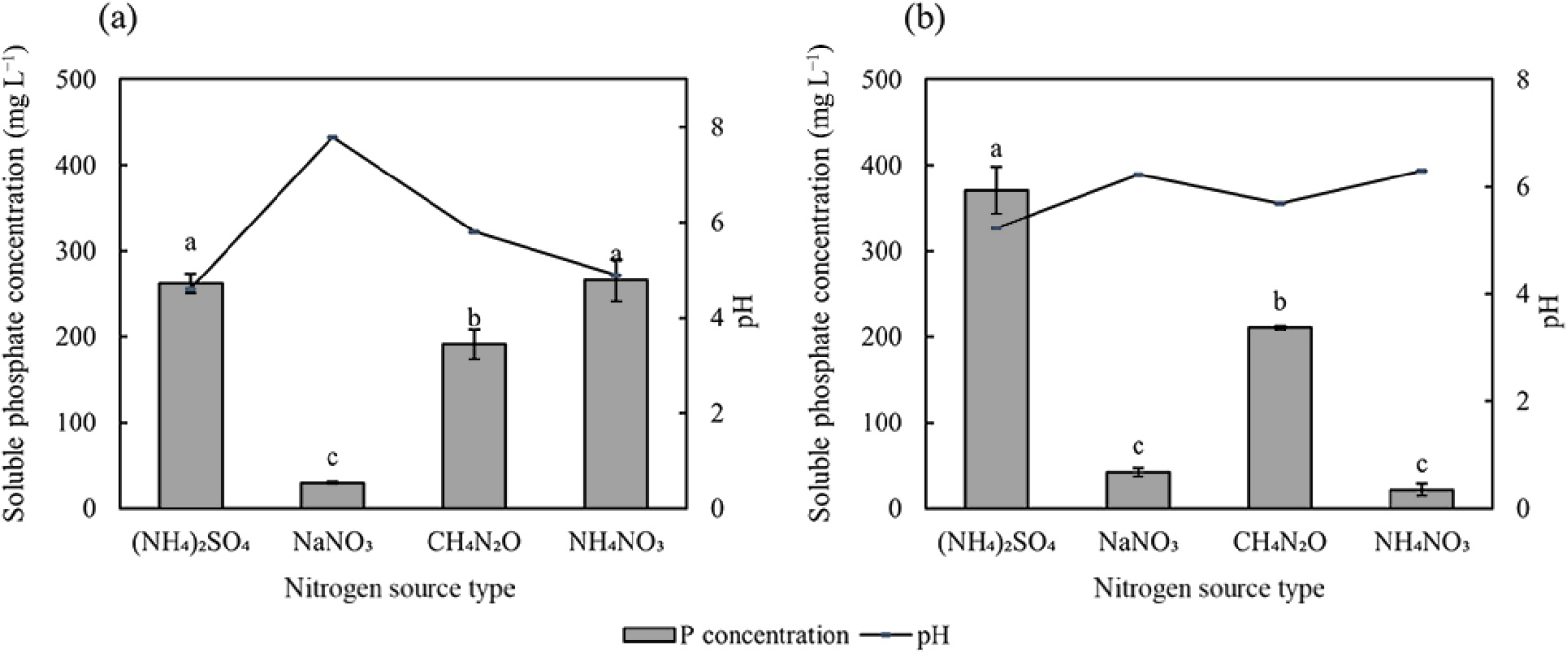
Effects of the nitrogen source on soluble phosphate concentrations after the cultivation of PSB2 (a) and PSB7 (b). The value of soluble phosphate shown in the figure is the amount of soluble phosphate in the control group that has been subtracted. Data are presented as means±SD. According to Tukey’s test, the same letters in the same column indicate no significant differences among treatments at a 0.05 level (*n*=3).

The nitrogen source also affected the amount of phosphate solubilized by PSB (Fig. 5). When ammonium nitrogen ([NH_4_]_2_SO_4_) was used as the nitrogen source, PSB2 and PSB7 both exhibited high phosphate solubilization capacities, with soluble phosphate concentrations in media of 262.10 and 371.13‍ ‍mg L^–1^, respectively. Additionally, the pH of media was significantly lower than in other treatment groups. Furthermore, PSB2 exhibited a high phosphate solubilization capacity when ammonium nitrogen and nitrate nitrogen (NH_4_NO_3_) were used as nitrogen sources. Neither strains effectively utilized nitrate nitrogen (NaNO_3_).

**Fig. 6.**
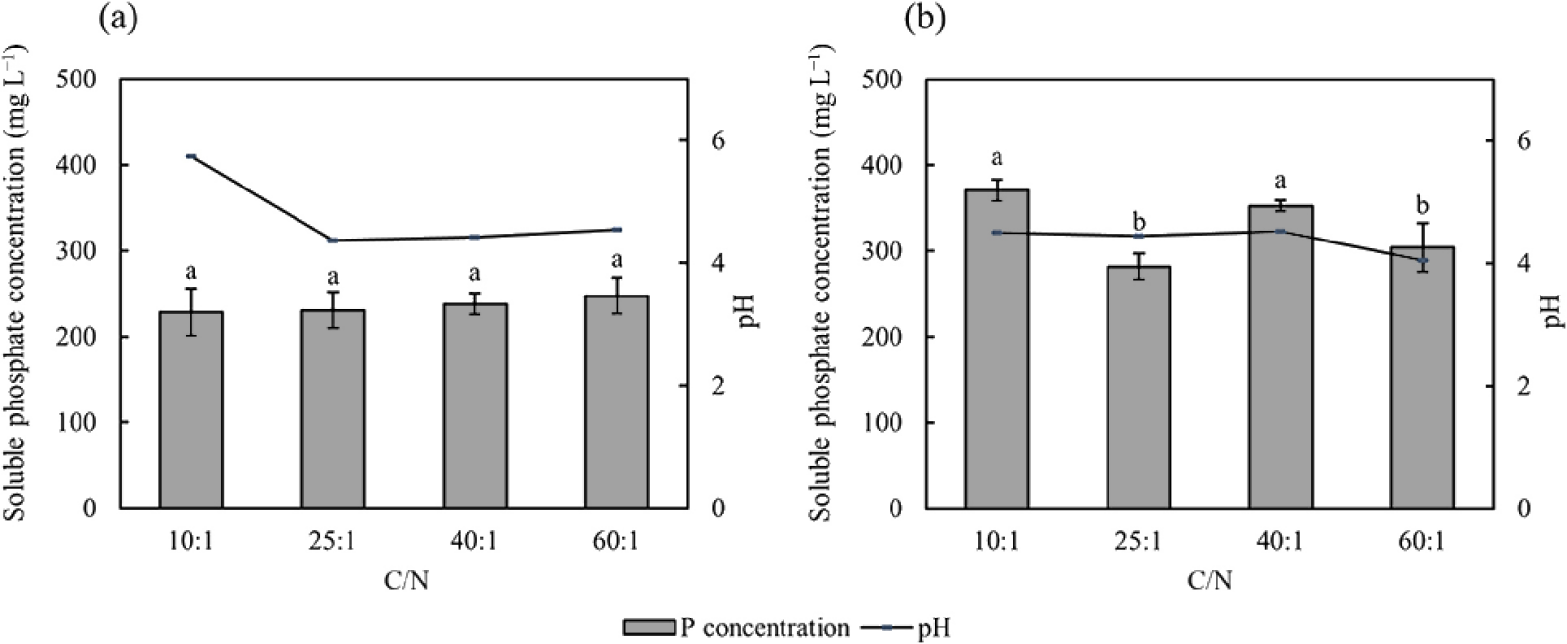
Effects of C/N on soluble phosphate concentrations after the cultivation of PSB2 (a) and PSB7 (b). The value of soluble phosphate shown in the figure is the amount of soluble phosphate in the control group that has been subtracted. Data are presented as means±SD. According to Tukey’s test, the same letters in the same column indicate no significant differences among treatments at a 0.05 level (*n*=3).

**Fig. 7.**
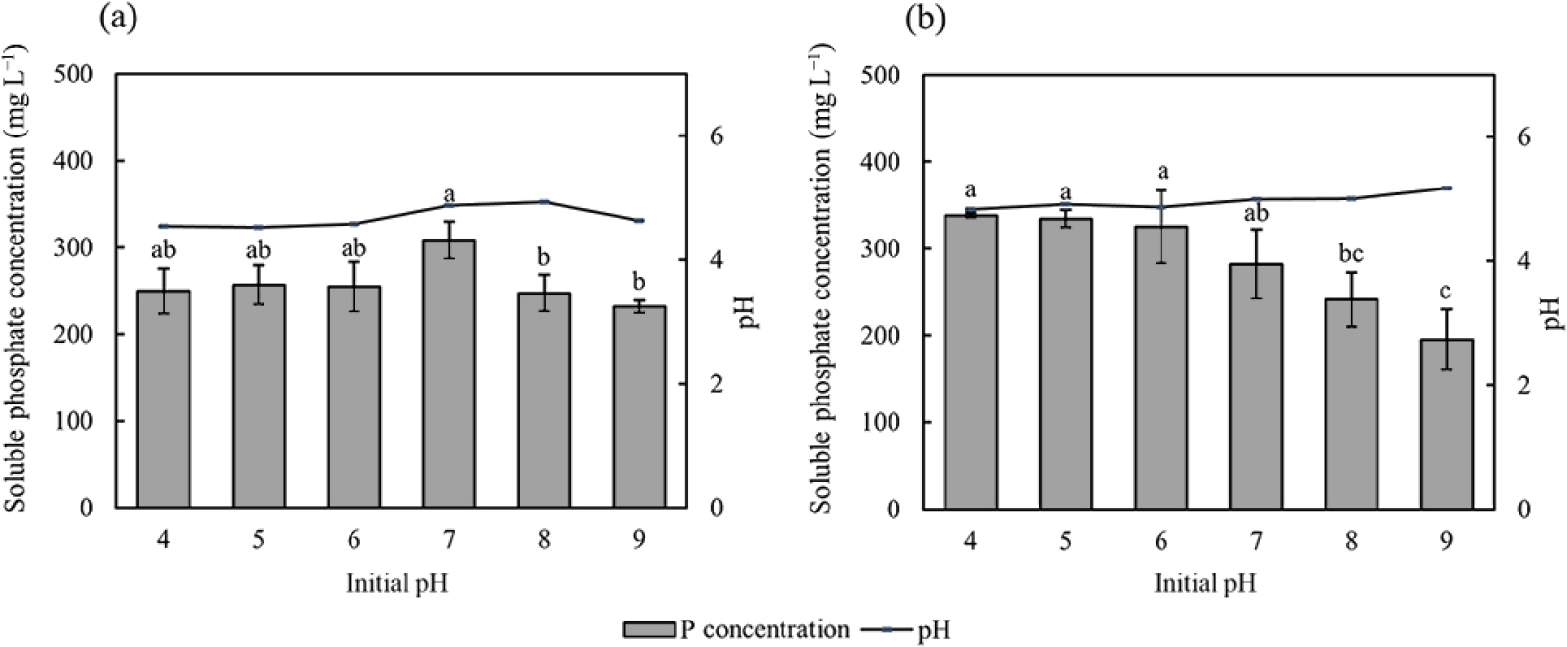
Effects of the initial pH on soluble phosphate concentrations after the cultivation of PSB2 (a) and PSB7 (b). The value of soluble phosphate shown in the figure is the amount of soluble phosphate in the control group that has been subtracted. Data are presented as means±SD. According to Tukey’s test, the same letters in the same column indicate no significant differences among treatments at a 0.05 level (*n*=3).

C/N affected the amount of phosphate solubilized by PSB7, but not by PSB2 (Fig. 6). When C/N was 10:1 or 40:1, the soluble phosphate concentration in the medium was higher than in other treatments. The initial pH affected the amount of phosphate solubilized by PSB (Fig. 7). When the initial pH was 7, the soluble phosphate concentration in the medium of PSB2 was the highest at 308.41‍ ‍mg L^–1^. Furthermore, as the initial pH in the medium decreased, the capacity of the bacterium to dissolve phosphorus increased. The phosphate solubilization capacity of PSB7 was the strongest when pH was 4, with a soluble phosphate concentration of 338.18‍ ‍mg L^–1^ in the medium. Conversely, the lowest amount of soluble phosphate dissolved by PSB7 occurred when pH was 9.

### Effects of PSB inoculations on plant phosphorus nutrition

As shown in Table 1, the addition of PSB2 increased the phosphorus content in plant roots, while the addition of PSB7 increased the phosphorus content of the entire plant. Additionally, a significant increase was observed in the biomass of plant roots. However, no significant changes were detected in the phosphorus concentration available in soil or alkaline phosphatase activity. The addition of PSB7 to the soil facilitated phosphorus absorption by plants more effectively than that of PSB2.

## Discussion

Few studies have focused on the isolation and identification of PSB in the Loess Plateau. The present study screened for PSB with high phosphate solubilization capacities in the rhizosphere of locust forests on the Loess Plateau, with the aim of filling some gaps in PSB research. The results obtained showed that the amounts of phosphate solubilized in cultures of PSB2 and PSB7 after 6 days were significantly higher than those of other strains: 9.68-fold and 11.61-fold higher, respectively, than that of the control group. Additionally, the pH of their cultures was significantly lower than those of the other strains. A low pH in their culture media is evidence for their strong phosphate solubilization capacities because they invariably secrete organic acids during their growth and metabolism (Miller *et al.*, 2010; Billah *et al.*, 2019). These acids are the main force dissolving calcium phosphate (Fig. 1).

A phylogenetic ana­lysis of the 16S rRNA gene indicated that strain PSB2 belonged to *Pseudomonas* and PSB7 to *Inquilinus* (Fig. 3). *Pseudomonas* is one of the most common types of PSB (Panwar *et al.*, 2014). Within this genus, phosphate solubilization capacities vary among different species. Gupta *et al.* (2012) and Tomer *et al.* (2017) conducted tests on the phosphate solubilization capacities of *Pseudomonas* isolated from soil. Even though the species tested were all *Pseudomonas*, the concentrations of phosphorus released ranged between 14.49 and 398.14‍ ‍mg L^–1^, which markedly differed. Based on these findings, the phosphate solubilization capacity of PSB2 was excellent. As shown in Fig. 2, *Inquilinus* exhibits a close genetic relationship with *Azospirillum*, a well-known “plant-growth promoting rhizobacteria” (PGPR) (Ayyaz *et al.*, 2016). In comparisons with *Pseudomonas*, *Inquilinus* represents a newly discovered species that may serve as a plant PGPR (Kaplan *et al.*, 2019). Since Jung *et al.* (2011) identified *Inquilinus ginsengisoli*, only a few studies have found bacteria with similar rhizospheric growth-promoting benefits (Rangel *et al.*, 2017; Man *et al.*, 2021). To the best of our knowledge, no studies have isolated *Inquilinus* as PSB from calcareous soil in the Loess Plateau.

Changes in the carbon source, nitrogen source, and initial pH of the NBRIP liquid medium significantly affected the ability of PSB2 and PSB7 to dissolve phosphorus. Changes in growth conditions resulted in distinct phosphate solubilization characteristics exhibited by the two strains selected in this study. PSB exhibited robust phosphate solubilization capacities when glucose was used as the carbon source (Fig. 4), which is consistent with the findings of Hameeda *et al.* (2006). These bacteria secrete a higher quantity of the aldonic acids, 2-ketogluconic acid and gluconic acid, which may be one of the main reasons for this phenomenon because these acids chelate metal cations, leading to the release of phosphate ions (Miller *et al.*, 2010; Oteino *et al.*, 2015).

PSB2 exhibited the strongest phosphate solubilization capacity when ammonium nitrogen was used as the nitrogen source (Fig. 5), which is consistent with the findings by Prabhu *et al.* (2018). Since microorganisms harness energy from the conversion of ATP during NH_4_^+^ assimilation, they subsequently release H^+^ through a proton pump to lower pH. This acidification process facilitates the dissolution of phosphorus (Ahuja *et al.*, 2007). However, PSB7 exhibits distinct behavior. Its phosphate solubilization capacity in the presence of ammonium nitrogen and nitrate nitrogen was significantly lower than when only ammonium nitrogen was present in the medium. This may be attributed to a reduction reaction during the bacterial assimilation of NO_3_^–^, leading to the release of OH^–^ and subsequent increase in pH (Nahas, 2007; Sharan *et al.*, 2008). Consequently, this elevation in pH results in a decrease in soluble phosphate concentrations (Du *et al.*, 2019).

A correlation was observed between the pH of the medium supernatant and the efficiency of phosphorus dissolution; however, it was not linear (Marra *et al.*, 2015).‍ ‍Acids secreted by bacteria may reduce environ­mental‍ ‍pH, and excessively low pH conditions may also restrict their growth. In comparison with fungi, bacteria prefer to inhabit neutral or alkaline environments. Zeng *et al.* (2017) indicated that a neutral environment was more favorable for phosphate solubilization by *Pseudomonas frederiksbergensis*. In the present study, PSB2, a pseudomonad bacterium, similarly exhibited its optimal phosphate solubilization capacity when the initial pH of the culture medium was 7 (Fig. 7). On the other hand, PSB7 belongs to the genus *Inquilinus*, which represents a group of bacteria that thrive in acidic environments (Jung *et al.*, 2011). We also observed that decreases in the initial pH of the culture medium were associated with increases in phosphate solubilization capacity (Fig. 7). Therefore, bacteria appear to respond differently to the initial pH of the medium due to their different compositions and metabolic types. Furthermore, changes in C/N only affected the amount of phosphate solubilized by PSB7, but not by PSB2 (Fig. 6). This may be attributed to changes in the type and quantity of organic acids released by PSB7 under different C/N (Estrada-Bonilla *et al.*, 2017). Further research is needed to investigate the effects of different acid categories.

In the present study, inoculations with PSB significantly increased the phosphorus content in plant roots, but had no effect on the plant biomass. This may be related to the insufficient amount of the inoculum added to potted soil and the duration of the treatment after the PSB inoculation. Chen *et al.* (2021) exami­ned the growth-promoting effects of PSB on Chinese fir; the PSB inoculum was repeatedly added to soil and pot experiments were also conducted for up to three months. The findings obtained showed that PSB increased the plant biomass. In this pot experiment, the 1% (v/w) PSB‍ ‍inoculant was added only once. Additionally, *R. pseudoacacia* is a woody plant with a long growth cycle; therefore, the growth-promoting effects of PSB inoculations cannot be characterized in a short time period. An increase in the inoculum provides more bacteria for the dissolution of calcium phosphate, resulting in more available phosphorus being released. In addition, increasing the cultivation time gives plants more time to utilize phosphorus, which may be reflected in various characteristics, such as biomass, plant height, and ground diameter.

Furthermore, the concentration of available phosphate in the soil remained unchanged, whereas the content of phosphorus in plant roots increased. This result indicates that a‍ ‍dynamic equilibrium was reached in the soil-plant-microorganism system. At this phosphorus level, some soluble phosphate in the soil is fixed by calcium ions, while some is absorbed and stored by plant roots. Previous studies demonstrated that PSB proliferated normally and continued to decompose calcium phosphate (Zhang *et al.*, 2005; Harris *et al.*, 2006). It is also possible that hormones secreted by PSB during growth, such as auxins, cytokinins, and gibberellins, promote plant growth, as described by Puri *et al.* (2020). As plants grow faster, their ability to absorb phosphorus increases, resulting in the available phosphorus content in the soil being maintained at a certain level.

Therefore, the inoculations with PSB, particularly PSB7, significantly increased the phosphorus content in the roots of *R. pseudoacacia*. The present results suggest the potential of this type of PSB as a phosphorus biofertilizer for *R. pseudoacacia*. Further field experiments are needed to confirm the long-term effects of PSB inoculations on *R. pseudoacacia*.

## Conclusion

Two strains, PSB2 and PSB7 with efficient phosphate solubilization capacities were screened in the rhizosphere of *R. pseudoacacia*. The concentrations of dissolved phosphate in their culture media were 9.68-fold and 11.61-fold higher, respectively, than that of the control group. After its identification, PSB2 was classified as *Pseudomonas*, while PSB7 belonged to *Inquilinus*. Both strains exhibited optimal phosphate solubilization capacities when supplied with glucose and ammonium nitrogen. In addition, PSB2 preferred to survive under neutral conditions and PSB7 under acidic conditions. Pot experiments indicated that inoculations with PSB effectively increased the phosphorus content in the roots of *R. pseudoacacia*, with PSB7 exerting superior effects. The present results suggest the potential of PSB as a phosphorus biofertilizer for *R. pseudoacacia*. Additionally, they may be applied to facilitate soil and water management on the Loess Plateau.

## Citation

Zhang, W., Zhou, Y., Jia, J., Lu, Y., and Zhang, H. (2024) Isolation and Identification of Phosphate-solubilizing Bacteria in the Rhizosphere of *Robinia pseudoacacia* on the Loess Plateau and Verification of Phosphate Solubilization Capacity. *Microbes Environ ***39**: ME24001.

https://doi.org/10.1264/jsme2.ME24001

## Figures and Tables

**Table 1.** Effects of PSB inoculations on plant nutrition.

Treatments	Biomass (g)			Phosphorus content (mg)		Soil soluble phosphate (mg kg^–1^)	Soil alkaline phosphatase (mg g^–1^ 24 h^–1^)
	Shoot	Root		Shoot	Root		
CK	0.94±0.16a	0.49±0.08b		0.70±0.01b	0.10±0.02b	15.71±1.54a	2.9247±0.2457a
PSB2	1.31±0.21a	0.84±0.15ab		0.82±0.05ab	0.40±0.09a	16.64±2.64a	3.8273±0.3540a
PSB7	0.86±0.21a	1.01±0.10a		0.84±0.14a	0.37±0.05a	16.52±0.61a	3.3553±0.0965a

Data are presented as means±SD. According to Tukey’s test, the same letters in the same column indicate no significant differences among treatments at a 0.05 level. (*n*=5)
